# Boreal Forest Fire: UAV-collected Wildfire Detection and Smoke Segmentation Dataset

**DOI:** 10.1038/s41597-025-05634-0

**Published:** 2025-08-13

**Authors:** Julius Pesonen, Anna-Maria Raita-Hakola, Jukka Joutsalainen, Teemu Hakala, Waleed Akhtar, Niko Koivumäki, Lauri Markelin, Juha Suomalainen, Raquel Alves de Oliveira, Ilkka Pölönen, Eija Honkavaara

**Affiliations:** 1 Department of Remote Sensing and Photogrammetry, Finnish Geospatial Research Institute, Espoo, 02150 Finland; 2 Department of Computer Science, Aalto University, Espoo, 02150 Finland; 3 Faculty of Information Technology, University of Jyväskylä, Jyväskylä, 40100 Finland; 4 School of Marketing and Communication, University of Vaasa, Vaasa, 65200 Finland

**Keywords:** Scientific data, Natural hazards, Computer science

## Abstract

Automated image-based wildfire detection suffers from a lack of open-access data, especially data with annotations. Our dataset targets the gap by providing human and computer vision foundation-model co-annotated images from an uncrewed aerial vehicle (UAV) perspective from Finnish boreal forest environments. The images and videos were collected at multiple prescribed burning events, and the data were used to successfully train wildfire detection models in our previous studies, proving their value for the task. The Boreal Forest Fire dataset contains three sections: images with bounding box annotations, video clips with labels and images with segmentation masks. Alongside the data, we have released code, ensuring that the data is simple to use.

## Background & Summary

Wildfires pose significant threats to human life, wildlife, and ecosystems worldwide, emphasising the need for more effective detection, monitoring, and response systems. Technological advancements have driven the development of automated machine learning and sensor-based wildfire detection methods. Uncrewed aerial vehicles (UAVs) have emerged as a promising tool for the early detection and monitoring of wildfires. A comprehensive fire management system integrating these technologies could enhance situational awareness, facilitate real-time data transfer, and support informed decision-making during firefighting efforts, ultimately reducing the damage caused by wildfires.

Deep learning (DL) methods have gained prominence in wildfire detection^1–3^, yet challenges remain due to insufficient training data. Fire detection relies on key indicators, mainly flames and smoke. While flames are easier to detect due to their distinct colour and rapid movement, they may be obscured or absent in smouldering fires^4^. Smoke, visible from greater distances and often preceding flames, is crucial for early detection^2^, though its varying opacity and resemblance to clouds or fog complicate detection^5,6^. Using both indicators can improve the reliability of the detection systems. Addressing these data-related challenges is essential for deploying DL-based UAV wildfire detection systems, necessitating the collection, evaluation and preprocessing of high-quality datasets.

Recent studies highlight the growing use of neural networks (NNs) in wildfire detection^7^. Neural network models applied for the task include Faster R-CNN, YOLO v3, and VGG-16, which have shown success^7^. Video-based fire detection that leverages classification, segmentation, and object detection further improves analysis. However, suitable training data for detecting early wildfire indicators remains scarce^8^, underlining the importance of comprehensive, accurately annotated datasets.

A limited number of open-access datasets exist with binary classification labels of smoke/non-smoke, facilitating the training of both detection and segmentation models. Existing datasets include FLAME^9^, Furg-Fire^8^, HPWREN^10^, DeepFire^11^, FIRESENSE^12^, and The Corsican Fire Database^13^. While these datasets provide annotations for classification, few support segmentation or object detection directly. Given the importance of both flames and smoke as fire indicators, a dataset addressing these gaps is crucial. The Boreal Forest Fire dataset^14^ aims to strengthen available data by providing bounding box annotations for smoke detection, segmentation labels, and curated video clips with smoke/non-smoke classifications. For those who seek to use flames as fire indicators, we recommend looking into our published videos. While not annotated frame-wise, much of the footage contains material that could be extracted, annotated, and used for flame detection and monitoring in wildfire and forest fire scenes.

**Fig. 1:**
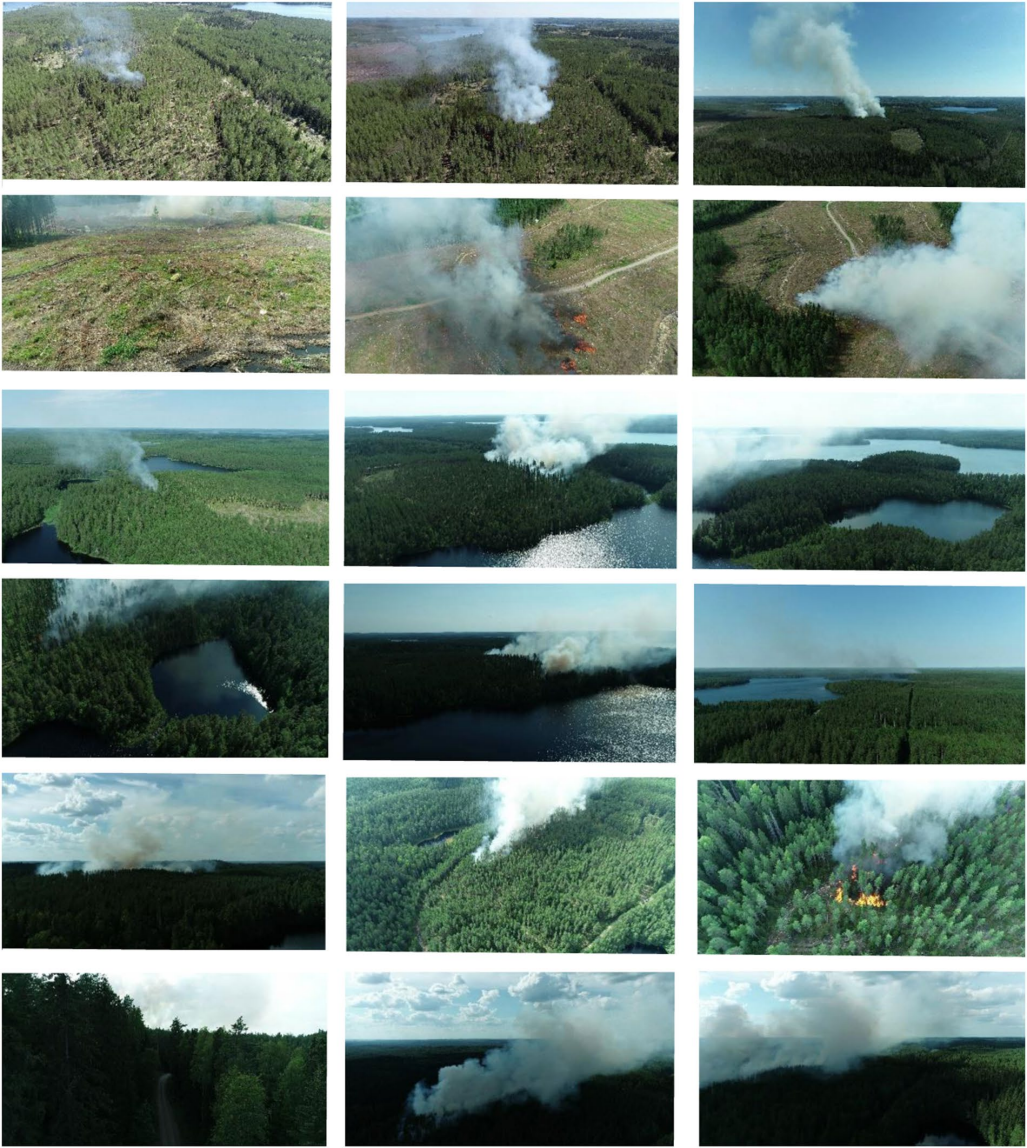
A Collage of Boreal Forest Fire dataset’s images highlighting the various angles, environmental features, and fire indicators found in the dataset. The collage is gathered from the Subset A images, including examples of all four locations.

The Boreal Forest Fire dataset^14^ is composed of 4954 images and 292 video clips collected from four locations in Finland during the boreal summer months. It includes human-annotated bounding boxes and semi-automatically generated segmentation masks using the Segment Anything Model (SAM)^15,16^ for smoke detection. Video clips were individually curated to support more accurate and generalisable DL applications for real-world fire monitoring. Besides the data itself, we published code samples for data visualisation and handling, and equations that ease the bounding box coordinate conversions between different object detection methods. Fig. 1 visualises the variability of the image scenes, as well as gives an overview of the boreal forest type and typical Nordic visual characteristics such as mirroring lake surfaces and cloudy sky.

## Methods

### Data collection

**Fig. 2:**
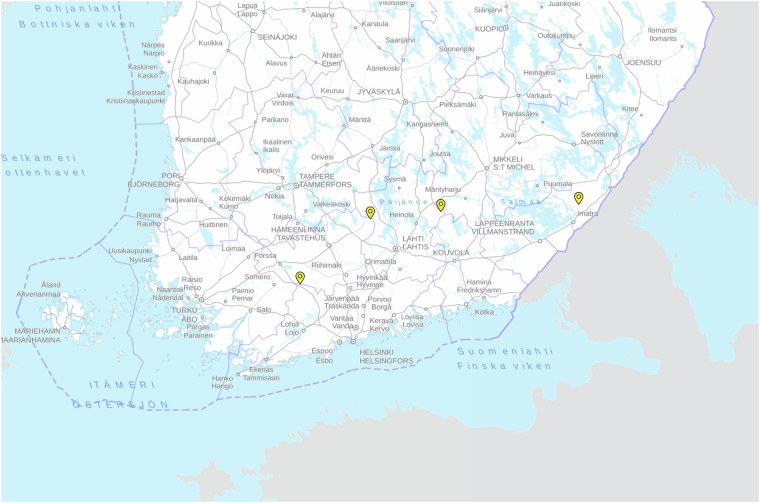
The smoke data collection locations are highlighted with yellow markers on top of a map of Finland^40^. The locations from west to east (left to right) are Karkkila, Evo, Heinola, and Ruokolahti.

The Boreal Forest Fire dataset^14^ was recorded during four forest restoration burnings in the summer of 2022. These controlled burns took place in four Finnish locations: Evo (E25.18555556, N61.22805556), Heinola (E26.44250000, N61.30083333), Karkkila (E23.97805556, N60.64222222), and Ruokolahti (E28.92222222, N61.35055556). The locations are shown on a map of Finland in Fig. 2. The burn sites varied in both weather conditions and terrain. The collection dates in the same order as the locations were May 24, June 28, July 6, and August 15, 2022.

**Fig. 3:**
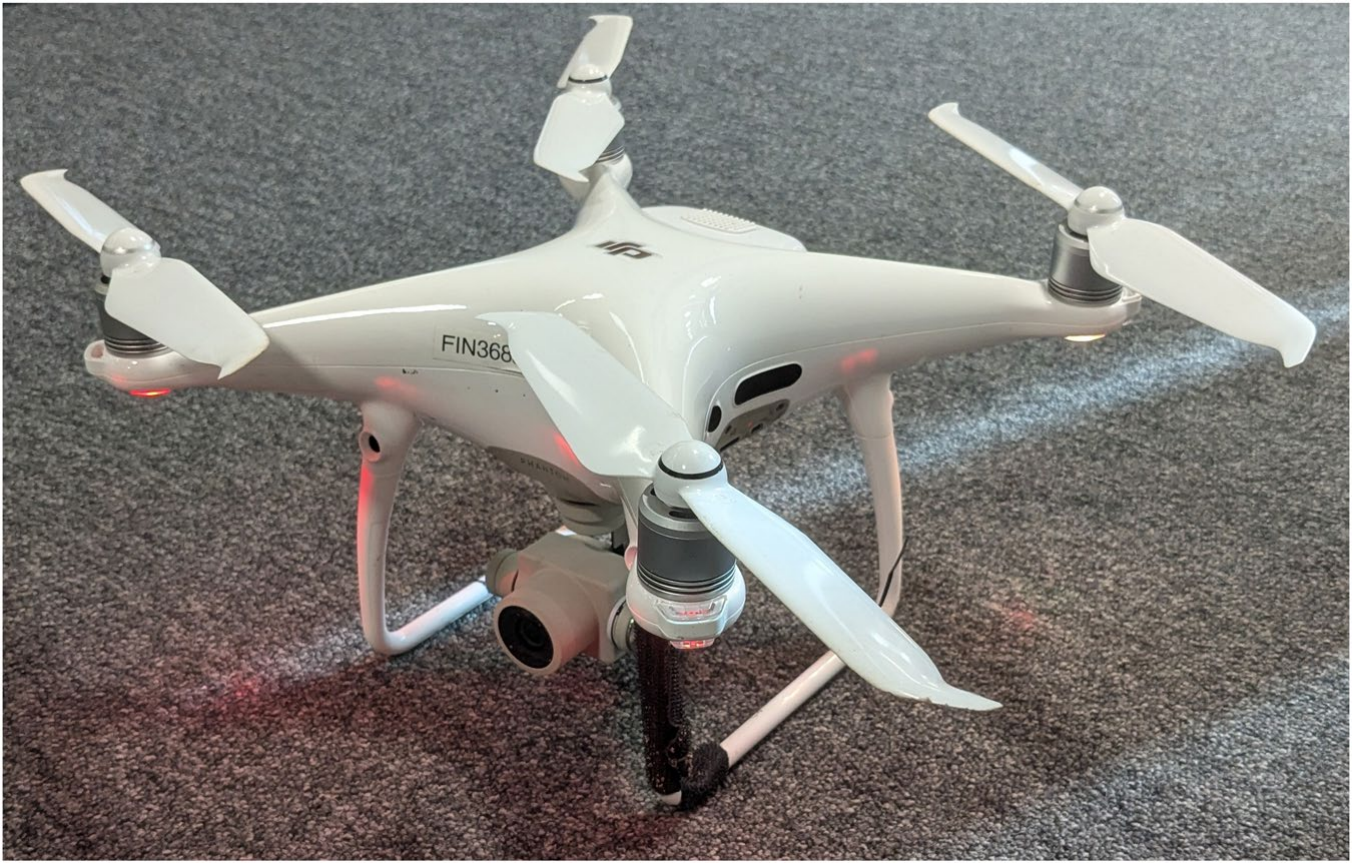
The DJI Phantom 4 UAV used for collecting the data.

The data collection was done using a DJI Phantom 4 UAV^17^. The UAV was used to capture RGB video with a 4K resolution (4096 × 2160 pixels). The used UAV is presented in Fig. 3. During the campaigns, the UAV flew between approximately 10 and 200 meters above ground and less than 500 meters away from the burning site, capturing the resulting smoke from various angles and distances at each event.

The dataset includes both close-range and long-range video footage. The camera was mostly pointed towards the burnings with the pitch angle of the camera varying from 0^°^ (horizontal) to −90^°^ (directly downwards). Fig. 1 presents a collage of the different angles of view, light and weather conditions, fire indicators, and a varying boreal forest type with water bodies, dry canopies, and tree species.

The dominant species of the forest in the imagery were Norway spruce and Scots pine, with smaller but visible quantities of silver birch and downy birch. The forest density and average age of trees also varied from one area to another. All observations were made in daylight, and the weather conditions were dry at each data-capturing date, but the cloud cover varied from completely clear to partly cloudy. The observed smoke clouds varied in shape, colour, and size due to varying environmental factors such as fire intensities, the burning vegetation, and wind conditions.

### Data preparation

#### Subset A with bounding box labels

The Boreal Forest Fire Subset A contains bounding box annotations, which were first introduced and used in our previous work^15^. The data was pre-processed with the following steps: The RGB images were extracted from MP4 videos and converted to JPG images with a Python script. With the script, we selected every 48th frame, corresponding to approximately one frame every two seconds. After extraction, the images were manually reviewed, and any frames containing artificial effects or elements subject to General Data Protection Regulation (GDPR) restrictions (such as identifiable individuals, vehicle license plates, or residential buildings) were removed.

The annotation strategy and the annotations were evaluated carefully. Since smoke is a difficult object for visual detection, we tested two annotation approaches. The first category contained large smoke annotations, where the bounding box was drawn around the smoke, including background information. The second category included several small bounding boxes, selected to contain only smoke in its various thicknesses. The images were manually annotated with the makesense.ai web tool^18^. After manually annotating all images and annotations of the Boreal Forest Fire dataset’s subset A, they were visually inspected image by image.

**Fig. 4:**
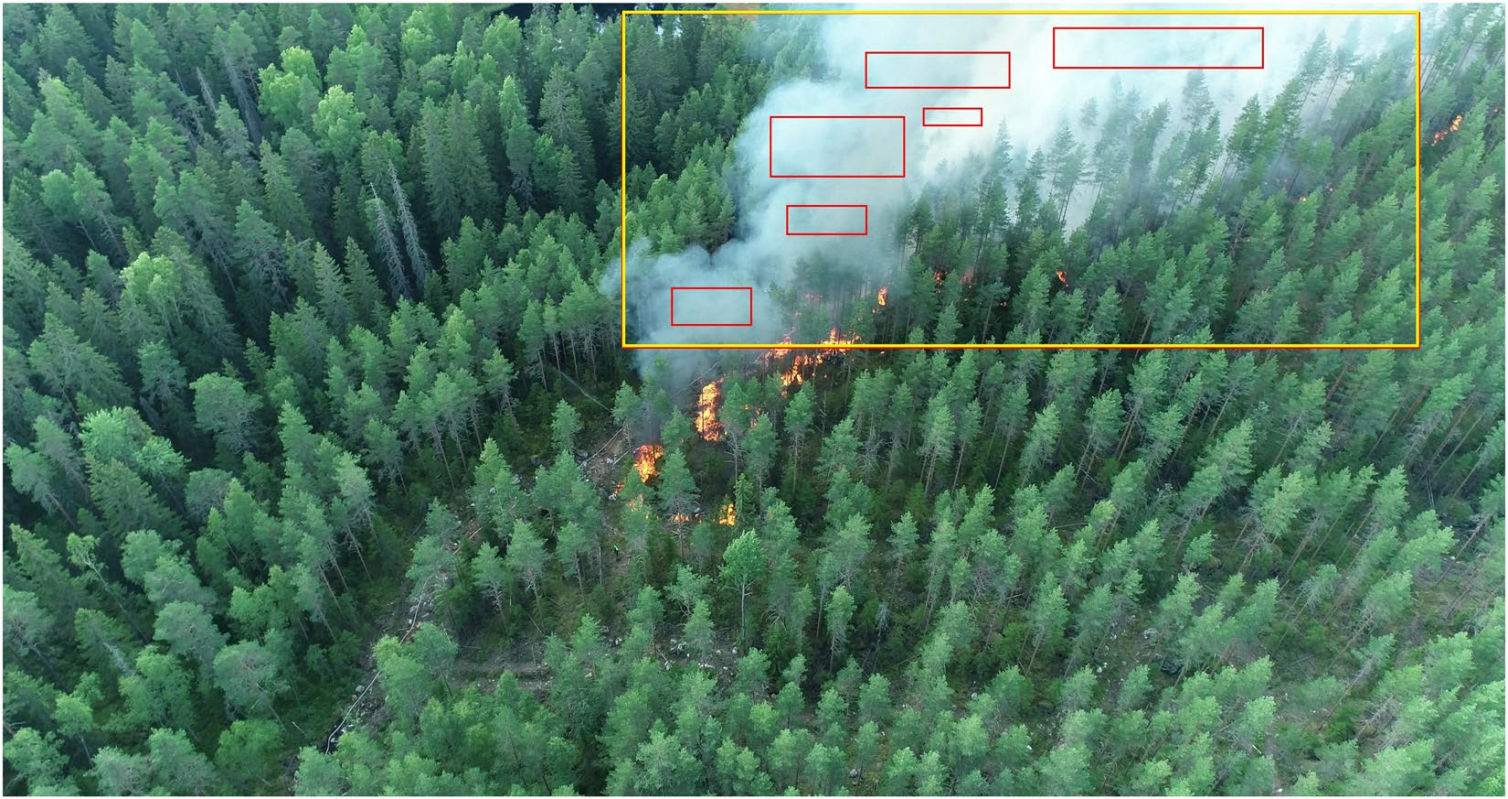
Boreal Forest Fire dataset annotation example from our earlier benchmark study^15^ showing the large bounding boxes (yellow) against multiple small bounding boxes (red). Only the large bounding boxes are included in the published data.

Due to the annotation strategy test results^15^, where the large smoke category (yellow box in Fig. 4) outperformed the smaller smoke annotations (red boxes), the smaller smoke annotations were removed from the database. The images and related labels (bounding box coordinates in text files) are organised in folders by location as shown in Fig. 8.

**Fig. 5:**
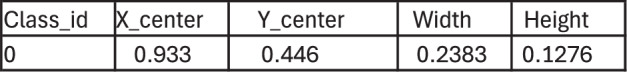
An illustration of a bounding box annotation.

**Fig. 6:**
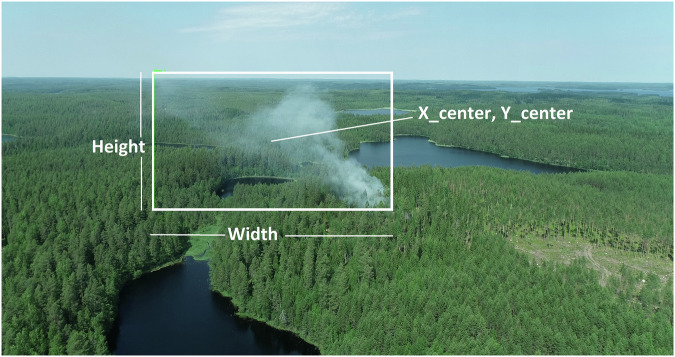
An example visualisation, where the annotation is drawn as a white bounding box.

The annotations of subset A follow the YOLO annotation format, shown in Figs. 5 and 6, consisting of images and the corresponding ground truth annotations. The YOLO format represents bounding boxes with normalised coordinates relative to the image dimensions. Each annotation file corresponds to an image and contains one entry per detected object.

Fig. 5 shows an example of the YOLO format coordinates, where integer **class_id** represents object binary categorisation, indicating which object is inside the bounding box coordinates. We only used a single “smoke” class, identified by a class label 0, to comply with the YOLO labelling convention. **X_center** and **Y_center** are normalised coordinates of the bounding box centre, meaning values between 0 and 1. Similarly, **Width** and **Height** are normalised values, describing the width and height of the bounding box.

#### Subset B with annotated video clips

Besides image-wise annotations, we prepared a collection of video clips which we suggest could be used in various ways to drive further research. As examples, those who wish to obtain more image data can extract and annotate new images from the videos. Another possibility is to use the clips as video streams in trials that show how image detection models behave qualitatively on video data or to test the inference speeds of DL-based object detection methods using small computational platforms, such as Raspberry Pi computers.

Before cutting the videos, the original drone footage was reviewed to remove non-purposeful or sensitive segments, such as identifying information, stationary ground-level footage, or any other irrelevant content. The videos were then segmented into fixed 30-second clips using the FFmpeg-based GUI tool, LosslessCut^19^, that was chosen to preserve the original video quality. The clips were then saved in MP4 format with indexed filenames indicating the location where the footage was captured, such as evo_1. Each video was given a corresponding ground truth label (boolean value), indicating whether the footage contains forest fire smoke, which was stored in a separate file.

#### Subset C with segmentation labels

**Fig. 7:**
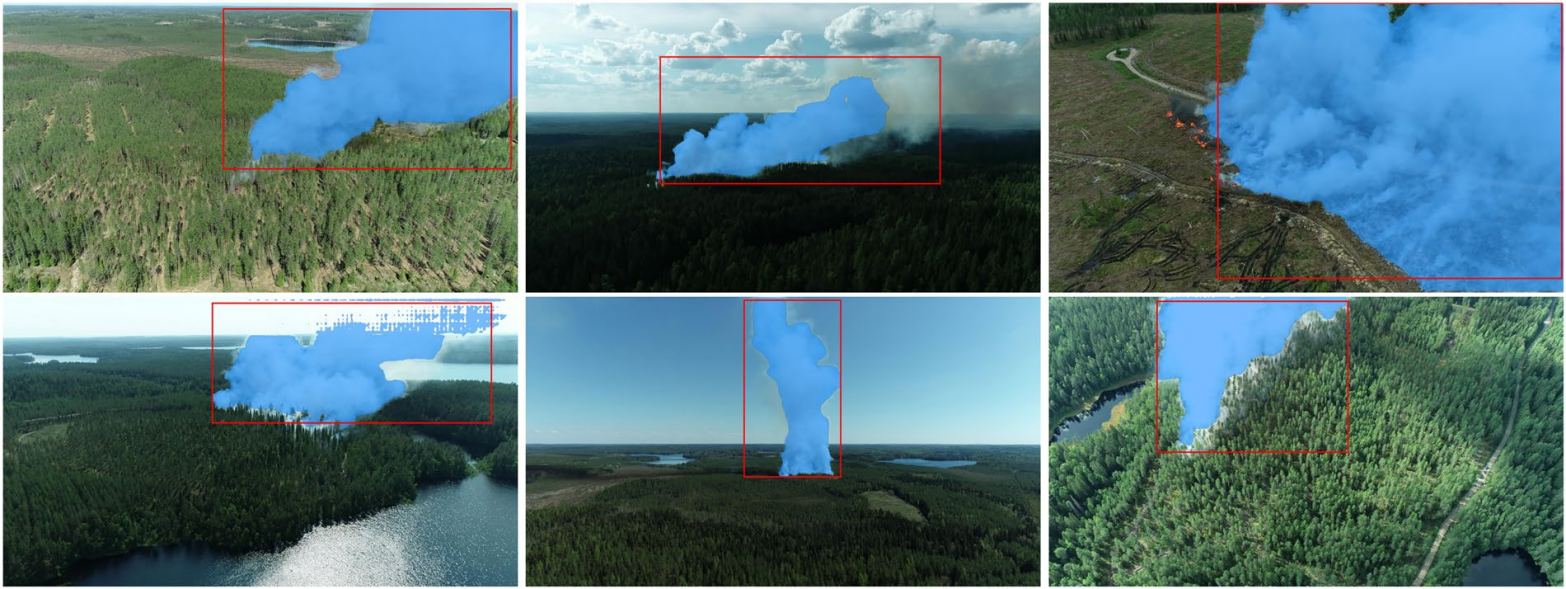
Sample images from the dataset with the SAM-generated masks shown in blue and the hand-annotated bounding boxes in red.

We also generated segmentation masks for the images containing smoke. For a small set of 40 images, these masks were drawn manually to serve as a test set for learning-based methods. For the rest, the masks were generated using the Segment Anything Model (SAM)^20^. SAM is a model that uses an image and a prompt in the form of a box or a point to generate a segmentation mask. To generate the smoke masks, the human-drawn bounding box and the original image were used as inputs, and the masks were stored as binary black-and-white PNG images. Examples of the annotated images are shown in Fig. 7.

## Data Records

**Fig. 8:**
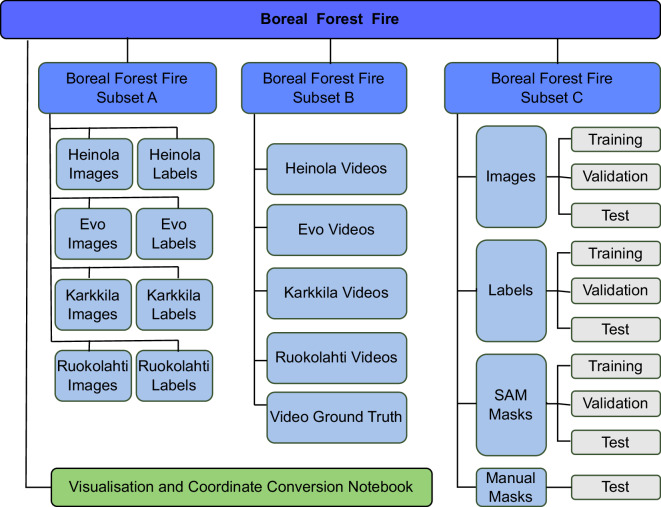
The data storage structure.

The dataset is available at Fairdata^14^. The Boreal Forest Fire dataset has three subsets. Subset A contains images and bounding box annotations. Subset B contains video clips and their corresponding labels, and Subset C contains images and segmentation masks. The corresponding annotation and image files are recognised by the structured naming convention, where the name before the file extension is the same for all corresponding files. The storage structure is visualised in Fig. 8.

### Subset A with bounding box labels

The Subset A contains 4954 images and the corresponding bounding box labels as text files. 256 images are empty; those are included with empty annotations (i.e. annotation files with no content). A total of 931 images were captured from Evo, 1767 from Ruokolahti, 1313 from Karkkila, and 943 from Heinola. The images and labels are stored in separate subdirectories based on the data-capturing locations.

### Subset B with annotated video clips

The Subset B contains 288 videos and binary, per-video annotations, with information on visible smoke in each video. A total of 31 videos were captured in Evo, 125 in Heinola, 74 in Karkkila, and 58 in Ruokolahti. The videos are stored in the original 4K, data-capturing resolution as MP4 files. The labels are stored in both a CSV and an Excel file. As per subset A, the videos are also stored in separate subdirectories based on their data-capturing locations, but the annotations are found in a single CSV or Excel file, stored in a separate subdirectory.

### Subset C with segmentation labels

For the segmentation study, a three-part datasplit, into training, validation and test sets, was performed. An approximately 120-second recording separation interval was used between frames to avoid major similarities between the sets. This resulted in a datasplit with 1184 images for training, 248 images for validation, and 40 images for testing. The difference in the total number of images between the two image subsets, A and C, was caused by images discarded due to large similarities.

The directory structure for the segmentation subset corresponds to the used datasplit. The images in the segmentation directory are also stored in the downsampled full HD (1080 × 1920 pixel) resolution used in the segmentation study. The naming of the files is consistent between the two subsets, making it possible to reconstruct the segmentation data in the original 4k resolution.

## Technical Validation

Our technical validation relies on our previously published, peer-reviewed studies that used data from Subsets A and C. In addition, we compared the dataset with other open-access data that have been used in wildfire detection and monitoring studies to highlight the need for new data.

### Images and bounding boxes (subset A)

The images were extracted in 2022 and first used in our earlier study^15^. The article concentrated on evaluating the features of smoke through annotation strategies and transfer learning and answered two research questions: *How general object is wildfire smoke in a visually strongly changing environment?* and *When we are using a pre-trained model, how much do we need locally collected data to generalise the wildfire model for fire detection in boreal forests?*

Regarding the quality of annotations. Smoke is a challenging object for visual detection due to its varying opacity, shape, and similarity to other natural elements like clouds or fog. In object detection, the choice of annotation strategy significantly impacts model performance, as bounding box size and quality influence training and evaluation outcomes. Bounding boxes are typically drawn as the smallest rectangle enclosing the entire object, but annotation is inherently subjective, especially when occlusions occur. Annotation fatigue can further degrade quality, leading to inconsistencies^7^. Therefore, the quality of two annotation strategies was evaluated: smaller bounding boxes in the same image representing only pure smoke and another class enclosing the entire smoke areas. However, as the smoke varies in shape, the bigger rectangles contain various environmental features besides the smoke clouds themselves. Our results confirmed that, for example, in all of the trials, the small smoke annotations resulted in significantly worse results: Using a YOLO v5 S model, fine-tuned with 1630 images with small annotations, the precision was only 0.24 and with larger annotations, the precision was 0.94^15^.

To evaluate generalisability and performance, we performed several tests using Karkkila, Evo and Heinola data, combined with HPWREN^21^ data, leaving Ruokolahti data for testing. Our results confirmed that the large annotations were sufficient for smoke objects, and smoke detection benefits highly from locally collected data. The model trained with the watchtower smoke images (HPWREN data) could not accurately detect smoke from Ruokolahti images. As an example, YOLO v5 L models, which were fine-tuned with HPWREN data, reached 0.93 precision with similar test data, but when the model was tested with Ruokolahti data, the highest precision of the same model architecture was only 0.031^15^.

YOLO v5 S, M, and L models all performed with higher precision, recall, mAP95, and mAP50-95 when the fine-tuning was done using local Boreal Forest Fire data^15^. The results of the YOLO study showed the technical quality of the annotations and the suitability of the images in smoke-based wildfire detection. It confirmed that locally collected data is required when the background of an opaque object, such as smoke, varies from other available training data. In addition, based on the study, a lightweight object detection model can achieve sufficient accuracy using relatively small amounts of high-quality data, suggesting that the size of our published dataset is adequate for many approaches.

### Videos (subset B)

We have provided the video clip subset mainly as the source of our labelled image datasets, with the idea that future users may find new ways to leverage as much of the original data as possible. Another possibility is that if needed, the users can select and extract frames with additional features, such as flames, for future annotation. In those cases, we recommend seeing the smoke-labelled clips, since smoke often appears with flames.

The video labels have not been tested for direct use in either image or video recognition model training or testing. The manually given labels are intended to serve as a way to ease the use of the video data in possible future studies. As such, the only technical validation that was done for the labels was a double-check of the label correctness by someone other than the one who created the video labels. Additionally, the quality of the data itself was validated by extension in the studies conducted for Subsets A and C, as the image data originated from these videos.

### Segmentation (subset C)

The segmentation data quality was validated in our previous study^16^ by training different PIDNet segmentation models^22^ with a mixture of our data and similarly labelled images from the Wildfire Smoke Dataset Versions 1.0 and 2.0 by AI For Mankind and HPWREN^23^. The quality of the SAM-generated masks was also quantified with the 40 manually annotated images, where the mean intersection over union (mIoU) of the labels was 0.636. In addition, pixelwise accuracy, precision, recall, and F1-score were measured, with values of 0.958, 0.912, 0.693, and 0.754, respectively. The measures show that the masks did not capture perfectly how a human perceives the smoke, and, namely, the low recall shows that the SAM-masks were more conservative in including smoke pixels in the masks. However, it’s worth noting that the bounding boxes were only used to guide SAM, but not the person drawing the manual masks, meaning that any differences in how the smoke was perceived by the person drawing the bounding boxes and the one drawing the pixelwise masks would also reflect on these measures.

In the practical tests, the trained segmentation model was able to detect a small smoke cloud from a prescribed forest burning event from up to 9.7 kilometres at pixel-level precision. The models were also tested on manually labelled images from both datasets to verify the quality of the resulting models. Finally, the mask generation method was compared to another solution where the masks were generated by a larger segmentation model, such as a Mask R-CNN^24^ with feature pyramid network (FPN) extracted features^25^, and a ResNet^26^ or Swin Transformer^27^ backbone. The study showed that the SAM-generated masks were comparable to the best fine-tuned models, and the SAM-based method was preferred due to its efficiency, which resulted from the fact that it did not require any retraining on the task. The best-performing of the trained PIDNet models trained using the SAM-generated masks was also the one validated at the real-world event with great results, showing that the data can be used to produce methods suitable for practical settings.

### Comparison with other similar datasets

Besides the Boreal Forest Fire dataset, we found the following six open-access datasets to be used for real-time fire detection in earlier scientific literature: FLAME^9,28^, Furg-Fire^8,29^, HPWREN Fire Ignition Library^10^, DeepFire^11,30^, FIRESENSE^12^, and The Corsican Fire Database^13,31^. We compared them to provide an overview of the technical specifications of different available datasets, with the chance of inspiring the readers to create models using more varied data from multiple sources instead of relying on limited samples. All the evaluated datasets provide predominantly feature annotations for classification, with only a few offering segmentation or object detection labels.

#### FLAME

The FLAME (Fire Luminosity Airborne-based Machine Learning Evaluation) dataset, published in 2020, is available on the IEEE Dataport^28^. Collected via drones during a prescribed burn in an Arizona pine forest, it includes ten data types, such as frames, masks, videos, and thermal heatmaps (infrared). The dataset features annotated frame-by-frame videos for flame-based fire detection in four spectral palettes: normal, white-hot, fusion, and green-hot.

As one of the first aerial wildfire datasets, FLAME supports aerial monitoring by offering varied resolutions, perspectives, and distances. Benchmark tests using NNs, U-Net, and segmentation methods have shown promising results in fire mask extraction and border detection^9^. Example studies using FLAME include^32–34^, and^9^.

#### Furg-Fire

The Furg-Fire dataset, published in 2016^29^, remains available but is no longer updated. It includes YOLO-annotated frames from various fire-scene videos, such as handheld, robot-mounted, and drone footage^8^. Designed for autonomous firefighter robots, its ground truth labels were created using OpenCV tools. The dataset includes benchmark tests and source code, but focuses on flame detection in diverse environments rather than wildfires. Example studies, such as^35^, apply data augmentation and tiny-YOLO v3 for real-time fire detection with promising results.

#### HPWREN Fire Ignition Library

The HPWREN camera network^21^ archives images from fixed-position cameras every minute, with the Fire Ignition Image Library (FIgLib) as a sub-collection focused on real fire scenes^10^. It includes hundreds of camera fire sequences, featuring colour, monochrome, and near-IR images. Data can be accessed via archive pointers or TAR file downloads. Some images have bounding box annotations, making them suitable for YOLO and Faster R-CNN detectors. Images captured 40 minutes before and after ignition support early smoke detection.

#### DeepFire

The DeepFire dataset was introduced with a benchmark study in 2022^30^, and the data is available on Kaggle^11^. The authors’ main idea was to create a diversified dataset from real-world forest imagery. The dataset is labelled for two classes, fire and no fire. The imagery contains frames that originate from different online sources. The fire-annotated images represent forests and mountains with visible flames or flames and smoke columns. A benchmark study with reasonable results was performed using a 80:20 train-test split^30^.

#### FIRESENSE

The FIRESENSE database, introduced in 2015, was developed for a project to protect cultural heritage areas from fire and extreme weather using multisensor networks^12^. It has been used, for example, in a study by Dimitropoulos *et al*.^36^. The database includes videos for flame and smoke detection, with binary classification for each: flames or no flames and smoke or no smoke.

#### Corsican database

The Corsican database, released in 2017^13,31^, is an evolving dataset and it contains annotated wildfire images in visible and near-infrared spectra. Each frame includes 22 parameters, such as spectral range, camera model, location, GPS coordinates, time of day, vegetation type, and cloud presence. While the dataset provides extensive metadata, the number of frames for teaching learning-based models is limited.

#### Dataset comparison

Table 1 summarises the key features of the six evaluated open datasets and the Boreal Forest Fire dataset. All datasets support classification with some ground truth, but gaps remain in available annotations. Segmentation and object detection annotations are rare, and most datasets focus on flames rather than smoke detection. Some datasets were not designed for wildfires but could be adapted for such applications. Only two datasets include bounding box annotations, with only HPWREN featuring wildfire scenes. Besides the evaluated open datasets, some fire detection datasets are also available upon request:^32,37–39^, and^5^.

**Table 1:** Comparison of dataset content types.

Dataset	Video	Video GT	Frames with labels	Classification	Segmentation	Object detection	Flame	Smoke	Smoke and Flame	Wildfire scene
FLAME	*✓*	*✓*	*✓*	*✓*	*✓*	—	*✓*	—	—	*✓*
Furg-Fire	*✓*	✗	*✓*	*✓*	—	*✓*	*✓*	—	—	✗
HPWREN	*✓*	*✓*	✗	*✓*	—	*✓*	—	*✓*	—	*✓*
DeepFire	✗	✗	*✓*	*✓*	—	—	*✓*	—	*✓*	*✓*
FIRESENSE	*✓*	*✓*	✗	*✓*	—	—	*✓*	*✓*	—	✗
Corsican database	✗	✗	*✓*	*✓*	*✓*	—	*✓*	*✓*	*✓*	*✓*
Boreal Forest Fire	*✓*	*✓*	*✓*	✗	*✓*	*✓*	✗	*✓*	✗	*✓*

Classification ground truth is available for all datasets.

As seen, our Boreal Forest Fire dataset strengthens the spectrum of available data by providing bounding box annotations, segmentation labels, and curated video clips with smoke information for developing and studying smoke detection models. Even though we have annotated only smoke, some of the images and videos also contain visible flames, leaving it possible to flexibly use them as fire indicators in the future.

## Usage Notes

### Jupyter notebook

We included a Jupyter Notebook file in our data release. The notebook contains easy-to-use code for visualisations (how to visualise bounding box annotations) and pixel coordinate conversion tools, which can be used to convert the bounding box format into other formats. To use the notebook, we recommend installing a Python environment and ensuring that the libraries called in the first cell of the code are properly installed. When loading the data from your computer, copy and paste the accurate file path into the notebook.

### Pixel conversions

Typically, some visualisation and object detection frameworks and libraries, such as Keras and Tensorflow, may require corner-based bounding box annotations and pixel conversion. The provided YOLO format can be transformed straightforwardly. For example, for an image of normalised dimensions (*W*, *H*), the pixel coordinates of the bounding box are computed as presented in Equations 1–3. The Python implementation of the conversion is found in the Jupyter Notebook.

1 $${x}_{center{\rm{\_}}new}={x}_{center}\times W$$

$${y}_{center{\rm{\_}}new}={y}_{center}\times H$$

$$width{\rm{\_}}new=width\times W$$

$$height{\rm{\_}}new=height\times H$$

Bounding box corners:

2 $${x}_{1}={x}_{center}-\frac{width}{2},\quad {y}_{1}={y}_{center}-\frac{height}{2}$$

3 $${x}_{2}={x}_{center}+\frac{width}{2},\quad {y}_{2}={y}_{center}+\frac{height}{2}$$

### Mixed datasets for segmentation

The segmentation model training and validation were performed with a mixture of the introduced dataset alongside the Wildfire Smoke Dataset Versions 1.0 and 2.0 by AI For Mankind and HPWREN^23^. The two datasets can be combined by simply merging the corresponding directories. The technical details and example code are available in the associated Gitlab repository: https://gitlab.com/fgi_nls/public/wildfire-real-time-segmentation.

## Data Availability

For visualisation of the dataset, a Jupyter Notebook is provided alongside this article. The notebook iterates over images of the dataset, finds corresponding annotations, conducts pixel operations and conversion from the YOLO format to bounding box corner coordinates, and finally displays a bounding box over the image via a pop-up window. This may be used as a basis for creating custom dataset utilisation strategies or understanding the data. The segmentation mask generation code and the associated model training and testing code are freely available on Gitlab, linked in Section Usage Notes. The code also includes visualisation tools for the segmentation data.
